# Transcriptional Regulation of the Equol Biosynthesis Gene Cluster in *Adlercreutzia equolifaciens* DSM19450^T^

**DOI:** 10.3390/nu11050993

**Published:** 2019-04-30

**Authors:** Ana Belén Flórez, Lucía Vázquez, Javier Rodríguez, Begoña Redruello, Baltasar Mayo

**Affiliations:** 1 Departamento de Microbiología y Bioquímica, Instituto de Productos Lácteos de Asturias (IPLA-CSIC), Paseo Río Linares s/n, Villaviciosa, 33300 Asturias, Spain; lucia.vazquez@ipla.csic.es (L.V.); bodesau@icloud.com (J.R.); baltasar.mayo@ipla.csic.es (B.M.); 2 Servicios Científico-Técnicos, Instituto de Productos Lácteos de Asturias (IPLA-CSIC), Paseo Río Linares s/n, Villaviciosa, 33300 Asturias, Spain; bredruel@ipla.csic.es

**Keywords:** equol, daidzein, isoflavones, transcriptional regulation, equol-producing bacteria, *Adlercreutzia equolifaciens*

## Abstract

Given the emerging evidence of equol’s benefit to human health, understanding its synthesis and regulation in equol-producing bacteria is of paramount importance. *Adlercreutzia equolifaciens* DSM19450^T^ is a human intestinal bacterium—for which the whole genome sequence is publicly available—that produces equol from the daidzein isoflavone. In the present work, daidzein (between 50 to 200 μM) was completely metabolized by cultures of *A. equolifaciens* DSM19450^T^ after 10 h of incubation. However, only about one third of the added isoflavone was transformed into dihydrodaidzein and then into equol. Transcriptional analysis of the ORFs and intergenic regions of the bacterium’s equol gene cluster was therefore undertaken using RT-PCR and RT-qPCR techniques with the aim of identifying the genetic elements of equol biosynthesis and its regulation mechanisms. Compared to controls cultured without daidzein, the expression of all 13 contiguous genes in the equol cluster was enhanced in the presence of the isoflavone. Depending on the gene and the amount of daidzein in the medium, overexpression varied from 0.5- to about 4-log_10_ units. Four expression patterns of transcription were identified involving genes within the cluster. The genes *dzr*, *ddr* and *tdr*, which code for daidzein reductase, dihydrodaidzein reductase and tetrahydrodaidzein reductase respectively, and which have been shown involved in equol biosynthesis, were among the most strongly expressed genes in the cluster. These expression patterns correlated with the location of four putative ρ-independent terminator sequences in the cluster. All the intergenic regions were amplified by RT-PCR, indicating the operon to be transcribed as a single RNA molecule. These findings provide new knowledge on the metabolic transformation of daidzein into equol by *A. equolifaciens* DSM19450^T^, which might help in efforts to increase the endogenous formation of this compound and/or its biotechnological production.

## 1. Introduction

Epidemiological and interventional studies suggest that soy isoflavones are beneficial to human health inasmuch as they are associated with lessened menopause discomfort in women [1]. They have also been related with a fewer reduced risk of suffering hormone-dependent, cardiovascular and neurodegenerative diseases, and certain types of cancer within general population [2,3,4,5]. In soy, isoflavones are found mostly as glycoside conjugates (daidzin, genistin, and glycitin) [6]. These β-glycosides have low estrogenic activity, and must be hydrolyzed into bioavailable isoflavone-aglycones (daidzein, genistein and glycitin, respectively) by cellular enzymes or enzymes from gut bacteria [7]. In the intestine, isoflavone-aglycones undergo further metabolic reactions generating compounds of greater biological activity or inactive metabolites [8]. Equol is a metabolite derived from the metabolism of daidzein, a major isoflavone predominantly found in soy-containing foods, produced exclusively by certain bacteria in the gut of humans and animals [9]. Equol is the isoflavone-derived compound with the strongest estrogenic [10] and antioxidant [11] activities. Based on its structural similarity to mammalian estrogens, equol may effectively bind to type β estrogen receptors but not those of type α, preventing menopausal symptoms without increasing the incidence of breast cancer [12]. All the animal species tested so far produce equol in response to soy or daidzein-containing diets [13]. However, it is produced by only 30–60% of humans [14,15]; it is likely that only these people will fully benefit from soy/daidzein consumption. This substantial inter-individual variation in equol production has been explained by differences in the composition of the gut microbiota [16].

Equol is an optically active molecule with two different enantiomers (R and S). However, the bacterial conversion of daidzein seems to produce only (*S*)-equol [13]. All but one of the equol-producing microbes isolated so far belong to the family Coriobacteriaceae [17]. Equol-producing strains have been identified for *Adlercreutzia equolifaciens* [18], *Asaccharobacter celatus* [19], *Enterorhabdus mucosicola* [20], *Slackia equolifaciens* [21], and *Slackia isoflavoniconvertens* [22]. Some other strains have been only identified at the genus level, and are named after their strain code, e.g., *Eggerthella* sp. YY7918 [23], *Paraeggerthella* sp. SNR40-432 [24], and *Slackia* sp. NATTS [25]. The single non-Coriobacteriaceae equol-producing strain identified so far is *Lactococcus garvieae* 20-92 [26]. Bacterial equol biosynthesis from daidzein proceeds via the intermediates dihydrodaidzein and tetrahydrodaidzein [27,28,29]. Gene cloning and genome analysis has revealed a gene cluster composed of eight open reading frames (ORFs) with a very similar genetic organization in all the strains studied so far, including *L. garvieae* [23,26,28,29]. Of these genes, three coding for a daidzein-dependent NADP reductase (converting daidzein into (*R*)-dihydrodaidzein) (*dzr*) a dihydrodaidzein reductase (converting (*R*)-dihydrodaidzein into *trans*-tetrahydrodaidzein) (*ddr*) and a tetrahydrodaidzein reductase (transforming *trans*-tetrahydrodaidzein to (*S*)-equol) (*tdr*) have been reported to be essential for equol production in *S. isoflavoniconvertens* [29]. These enzymes are induced by the presence of isoflavones, as demonstrated in both *S. isoflavoniconvertens* [29] and *E. mucosicola* [30]. However, neither the metabolic pathway involved in the metabolism of daidzein to equol nor its regulation are fully understood in any equol-producing species. Further knowledge of the control and regulation of the genes involved in equol production is required for its large-scale biotechnological production and for the design of strategies aimed to increase the endogenous production of equol.

The present study reports the transcriptional analysis, by reverse-transcribed PCR and real-time quantitative PCR, of the genes and intergenic regions of the equol cluster in *A. equolifaciens* DSM19450^T^. In the presence of daidzein, all genes in the cluster were overexpressed. Although no evidence of distinct mRNA transcripts was seen, differences in the expression level of several groups of genes flanked by terminator-like sequences of different strength were observed. These results provide the first insight into the expression patterns in this bacterium of the genes involved in equol production in the presence and absence of daidzein.

## 2. Materials and Methods

### 2.1. Bacterial Strain and Growth Conditions

*Adlercreutzia equolifaciens* DSM19450^T^, an equol-producing microorganism [18], was used as a prototype bacterium for studying the transcriptional regulation of equol biosynthesis. The strain was grown in Gifu anaerobic medium (GAM; Nissui Pharmaceutical, Tokyo, Japan) supplemented with 5 g/L arginine (Merck, Darmstad, Gemany) (GAM-Arg); 2% agar was added to the broth when a solid formulation was required. Cultures were incubated at 37 °C under strict anoxic conditions (10% H_2_, 10% CO_2_, and 80% N_2_) in a Mac500 work station (Down Whitley Scientific, Shipley, UK). For equol production, an overnight culture of the strain in liquid GAM-Arg was used as a seed culture to inoculate at 10% fresh GAM-Arg broth supplemented with different concentrations of daidzein (0, 50, 100, 150, and 200 µM) (Toronto Research Chemicals, Toronto, Canada). Bacterial growth was monitored at 2 h time intervals for 24 h, measuring the optical density at 600 nm (OD_600_) in a spectrophotometer. The results are presented as the mean ± standard deviation of four independent cultures.

### 2.2. UHPLC Analysis

Metabolites such as daidzein, dihydrodaidzein, and equol were identified and quantified by UHPLC using a reversed-phase Acquity UPLC™ BEH C18 1.7 μm column [31]. Samples (0.2 mL) were harvested from bacterial cultures every 2 h for 24 h, filtered through a 0.2 µm PTFE membrane (VWR, Radnor, PA, USA), and used directly in UHPLC analyses. Metabolite concentrations were estimated based on calibration curves prepared with known quantities of the corresponding standard compounds (all from Toronto Research Chemicals). Measurements were obtained for four independent cultures.

### 2.3. Nucleic Acid Extraction and cDNA Synthesis

Genomic DNA was extracted and purified after growth in GAM-Arg broth for 24 h using the DNeasy Blood and Tissue Kit (Qiagen, Hilden, Germany). Following the manufacturer’s recommendations for Gram-positive bacteria, but using an in-house lysis buffer (20 mM Tris-HCl, 2 mM EDTA, 1.2% Triton X-100, and 20 mg/mL lysozyme) supplemented with mutanolysin (0.1 U/µL) and RNAse (1.25 mg/mL). DNA was then eluted in sterile molecular biology grade water and stored at −20 °C until use. Total DNA was used as a template to determine the optimal amplification conditions and the efficiency of the primers used in succeeding reverse-transcription polymerase chain reaction (RT-PCR) and reverse-transcription quantitative PCR (RT-qPCR) assays.

Total RNA was obtained after growing the bacterium in GAM-Arg medium supplemented with daidzein (concentrations ranging from 50 to 200 µM), using a culture without the isoflavone as a control. Samples (5 mL) were harvested from the cultures during the exponential growth phase, corresponding to an OD_600nm_ of ~0.25 (approximately 8 h after inoculation). Cell pellets were obtained by centrifugation and stored at −20 °C until use. Total RNA was isolated from frozen pellets using the lysis method described above for DNA extraction, and purified using the RNeasy Mini Kit (Qiagen) according to the manufacturer’s instructions. Purified RNA was subjected to an additional treatment with DNase I (Qiagen) to eliminate any contaminating DNA. The absence of residual DNA in the samples was verified by real time PCR (qPCR) using the purified RNA as a template and the universal bacterial primers HDA1 and HDA2 of the 16S rRNA genes [32]. The concentration and purity of DNase-treated RNA samples were determined by measuring their absorbance at 260 nm (A_260_), and the ratio A_260_/A_280_ measured in an Epoch spectrophotometer (BioTek, Winooski, VT, USA). The purified RNA was stored at −80 °C until required for complementary DNA (cDNA) synthesis.

cDNA was produced from 0.25 µg of RNA using the iScript™ cDNA Synthesis Kit (Bio-Rad, Barcelona, Spain). Reverse transcriptase reactions were performed following the manufacturer’s instructions; i.e., one cycle of 25 °C for 5 min, 42 °C for 30 min and 85 °C for 5 min. The cDNA produced in this way was used as a template for qualitative and quantitative gene expression analyses.

Unless otherwise indicated, all the reagents employed in nucleic acid extraction and purification were purchased from Sigma-Aldrich (St. Louis, MO, USA).

### 2.4. Gene Expression Analysis

The qualitative expression of the genes within the equol cluster of *A. equolifaciens* DSM19450^T^ (Supplementary Table S1) was analyzed by RT-PCR. Based on the genome sequence for the strain deposited in the public NCBI database (GenBank Accession no.: GCA_000478885.1) [33], oligonucleotide primers within the ORFs from *AEQU_2235* through to *AEQU_2223* were designed (Supplementary Table S2). For better comparison of the transcriptional signals, primers were designed to produce amplicons of around 500 bp. RT-PCR was performed using the Taq DNA Polymerase Master Mix Kit (Ampliqon, Odense, Denmark), adhering to the following amplification protocol: an initial step at 94 °C for 5 min, followed by 33 cycles of 94 °C for 30 s, 55–68 °C (depending on the primer pair; Supplementary Table S2) for 30 s, and 72 °C for 45 s, plus a final extension step at 72 °C for 7 min. Expression levels of the 16S rRNA genes were used as controls to confirm there were no major differences in cDNA concentration between samples. The RT-PCR products were separated by electrophoresis in 2% agarose gels, stained with ethidium bromide (0.5 µg/mL), and visualized under UV light using a G Box Chemi XRQ gel doc system (Syngene International, Bangalore, India).

To elucidate the transcriptional organization of the above mentioned ORFs, new primers were designed within upstream and downstream ORFs to amplify the sequences of the complete intergenic regions (Supplementary Table S3). The PCR amplification conditions were the same as above. Additionally, the presence of inverted repeat sequences with a secondary structure that might provide transcriptional terminators was investigated using DNAMAN^®^ v.5.2 software (Lynnon Biosoft, San Ramon, CA, USA).

For selected genes, expression was quantified by RT-qPCR. Amplification was performed in an ABI PRISM 7500 thermocycler (Applied Biosystems, Foster City, CA, USA) using SYBR Green PCR Master Mix (Applied Biosystems). To ensure qPCR product size uniformity (≤90 bp) and melting temperature (60 °C ± 1 °C), primers (Supplementary Table S4) were designed using the algorithms provided by Primer Express v.2.0 software (Applied Biosystems). The efficiency of the primers was calculated based on the slope of a standard curve (Supplementary Table S4) and their specificity confirmed by detecting a single peak in the dissociation curve analysis of the amplicons. RT-qPCR was performed in triplicate for each target gene, with two independent experiments. To avoid variation among the samples in terms of the quantity and quality of cDNA, the expression of the housekeeping genes of *A. equolifaciens* glyceraldehyde-3-phosphate-dehydrogenase (GADPH) and the elongation factor Tu (EF-Tu) were used as controls. The results were recorded as the differential expression of a given gene with respect to the control sample (bacterial cultures grown without daidzein); i.e., by the 2^−ΔΔCt^ method.

## 3. Results

### 3.1. Growth of A. equolifaciens DSM19450^T^ with Daidzein

**Figure 1:**
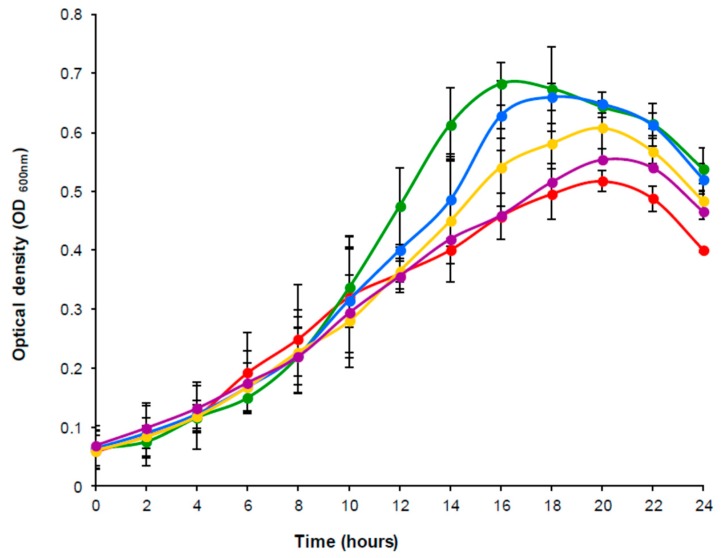
Growth kinetic curves of *A. equolifaciens* DSM19450^T^ grown in the presence of 50 µM (green), 100 µM (blue), 150 µM (yellow) and 200 µM (purple) of daidzein as compared to a control culture without daidzein (red).

The effect of daidzein on the growth of the bacterium was checked in parallel cultures grown in GAM-Arg supplemented with different concentrations of daidzein (0, 50, 100, 150, and 200 µM). Figure 1 shows the growth curves obtained under the given conditions over 24 h of incubation. No major differences were observed in the shape of the curves. Indeed, the profiles were almost identical until 10 h, after which, depending on the daidzein concentration, maximum growth occurred at 16 to 20 h of incubation, declining sharply thereafter. Compared to the control without daidzein, bacterial growth increased in the presence of all concentrations of daidzein tested (Figure 1). However, the maximum growth was reached in the culture supplemented with the lowest daidzein concentration (50 µM).

### 3.2. Metabolism of Daidzein by A. equolifaciens DSM19450^T^

**Figure 2:**
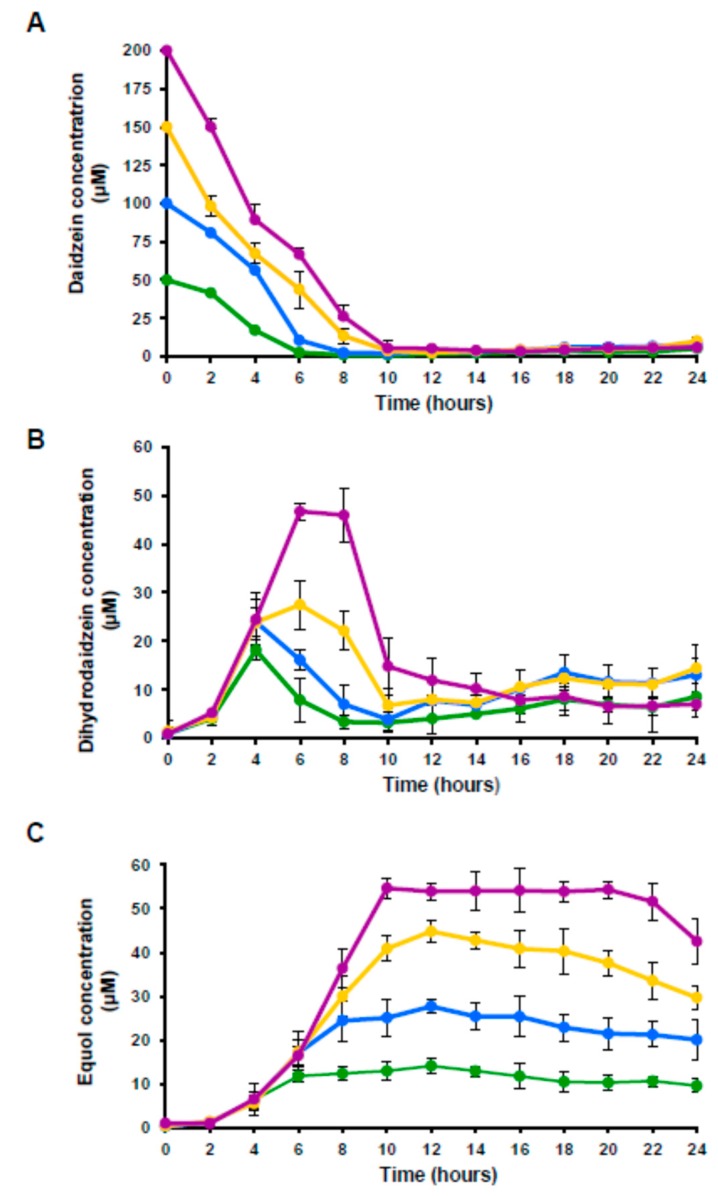
Evolution of daidzein (**A**), dihydrodaidzein (**B**), and equol (**C**) in cultures of *A. equolifaciens* DSM19450^T^ supplemented with 50 µM (green), 100 µM (blue), 150 µM (yellow) and 200 µM (purple) of daidzein.

The fate of the daidzein in the cultures, and its subsequent conversion into dihydrodaidzein and equol was followed over 24 h of incubation by UHPLC. Daidzein, dihydrodaidzein and equol were never detected in the absence of the isoflavone. Figure 2 shows the change in these three compounds over cultivation with the different amounts of daidzein. Depending on the initial amount, daidzein disappeared from the cultures between 6 and 10 h of incubation (Figure 2A). Dihydrodaidzein, which was monitored in parallel in the same cultures, showed maximum peaks at 4 to 6 h of incubation, reaching concentrations of 18, 24, 28 and 47 µM in the cultures with 50, 100, 150 and 200 µM of daidzein, respectively (Figure 2B). After this maximum, the dihydrodaidzein concentration decreased sharply after 10 h of cultivation, remaining thereafter constant at 5–10 µM. Equol reached their highest levels at about 10–12 h of incubation (Figure 2C), simultaneous with the stabilization of the dihydrodaidzein concentration; then, they remained constant until 24 h of incubation (Figure 2B). The maximum amount of equol produced was 14, 28, 42 and 54 µM for the cultures supplemented with 50, 100, 150 and 200 µM of daidzein respectively.

### 3.3. Identification of Daidzein-Induced Genes

**Figure 3:**
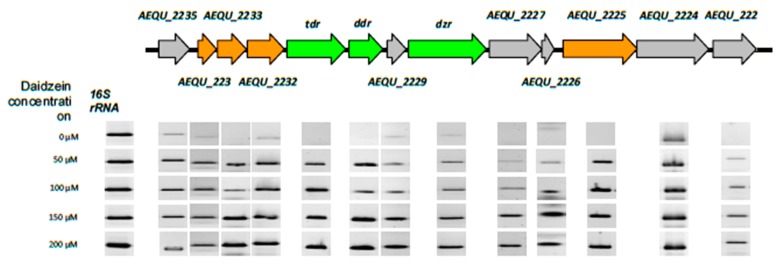
Schematic organization and RT-PCR analysis of genes in the equol biosynthesis cluster of *A. equolifaciens* DSM19450^T^. Color key: in green, genes proved to be involved in the conversion of daidzein to equol, as reported for *S. isoflavoniconvertens* [29]; in orange, genes with putative activity in daidzein metabolism; in gray, other genes. The band intensity represents the level of transcription of each individual gene in the presence of the different daidzein concentrations tested (on the left). As a control, the expression of the 16S rRNA genes was used.

Comparison of the ORFs from the equol biosynthesis clusters of *S. isoflavoniconvertens* and *A. equolifaciens* showed a high degree of linear conservation of genes, although identity at the deduced amino acid level ranged between 39% and 82% (Supplementary Figure S1). To identify the gene products involved in daidzein metabolism, gene expression analysis of all the ORFs in the equol operon was performed by RT-PCR. For this, total RNA was isolated from exponential-phase cultures without daidzein (control) and with the different amounts of daidzein stated above. Transcription of the 16S rRNA gene was identical in all cultures, indicating that variations in gene expression due to differences in cDNA concentration or quality could be ruled out (Figure 3). In the absence of daidzein, no transcription of the genes was detected or was significantly lower than in cultures with daidzein (except for ORF *AEQU_2224*, which showed a similar pattern of expression with and without the isoflavone) (Figure 3). Further, the expression of ORFs between *AEQU_2225* and *AEQU_2233*, which includes *tdr*, *ddr* and *dzr*, became higher with increasing daidzein in the culture medium, with the strongest expression recorded for 200 µM.

### 3.4. Transcriptomic Analysis of the Equol Gene Cluster in the Presence of Daidzein

**Figure 4:**
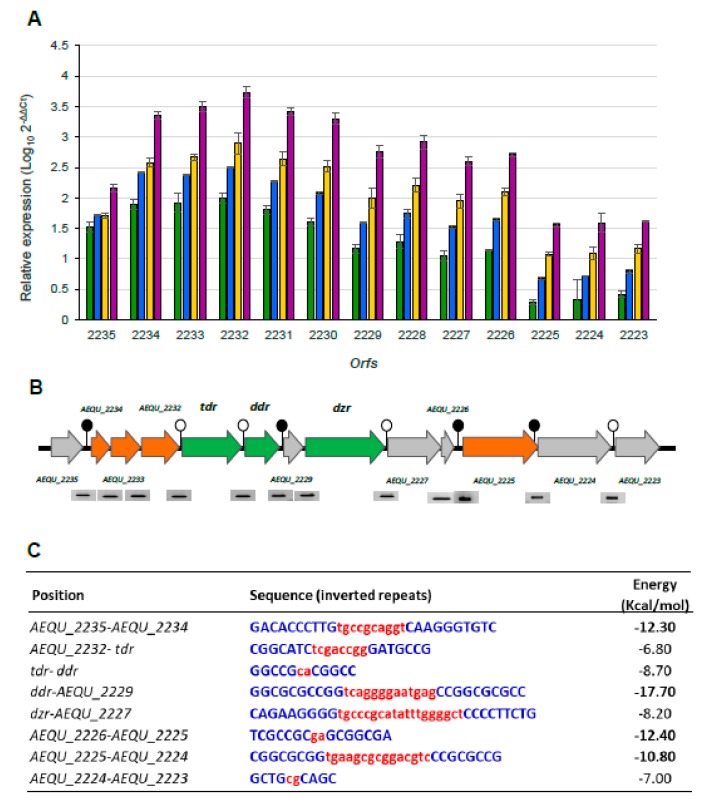
Quantification of the effect of daidzein on the expression of genes involved in the equol biosynthesis gene cluster of *A. equolifaciens* DSM19450^T^ by RT-qPCR (**A**). The graph shows the relative expression of the genes in the presence of 50 (green), 100 (blue), 150 (yellow) and 200 (purple) of daidzein in relation to gene expression of the reference condition (without daidzein) and after normalization with the expression of the elongation factor Tu housekeeping gene. Gene organization and transcriptional analysis by RT-PCR of the intergenic regions of the equol biosynthesis cluster of *A. equolifaciens* (**B**). Summary of the inverted repeat sequences resembling ρ-independent terminators and their respective free energy (kcal/mol) (**C**). Terminators with the lowest (in bold) and highest free energy are indicated by filled and unfilled circles, respectively, in Panel B.

RT-qPCR analysis was performed to quantify the effect of daidzein on the expression of ORFs in the equol operon. In the absence of daidzein, a very low basal expression of all the genes was recorded. Figure 4A shows the relative expression of each gene (with respect to the controls) in the presence of the tested daidzein concentrations (previously calibrated against EF-Tu expression). Highly similar results were obtained when expression was calibrated against the expression of the GADPH gene. In general, the quantification of the RT-qPCR transcripts corroborated the RT-PCR findings; i.e., the expression of all ORFs from *AEQU_2235* to *AEQU_2223* increased with increasing daidzein concentration (Figure 4A). However, these genes were not all overexpressed equally; indeed, four differential expression patterns were noted. The group of ORFs from *AEQU_2225* to *AEQU_2223* showed the lowest relative increases (up ~3.1–31.6 fold). A second expression pattern was recorded for ORF *AEQU_2235*, the relative overexpression of which (compared to controls) ranged between ~31.6 and 177.8-fold. A third expression pattern was recorded for ORFs between *AEQU_2229* and *AEQU_AEQ2226* (up ~17.7–1,000-fold), among which the *dzr* gene is included. Finally, the group of ORFs from *AEQU_2234* to *AEQU_2230*, which includes *tdr* and *ddr*, showed the highest level of overexpression with increases from ~100 to 10,000-fold (2 to 4 log_10_ units) at the lowest and highest daidzein concentration, respectively.

### 3.5. Transcriptional Organization of the Equol Biosynthesis Gene Cluster

In silico analysis of the intergenic sequences in the equol biosynthesis gene cluster revealed eight putative ρ-independent terminator-like sequences in the intergenic regions. Four of the eight predicted terminators consisted of stems of 7–10 nucleotides separated by loops of two to fifteen nucleotides, upstream of the ORFs *AEQU_2234*, *AEQU_2229*, *AEQU_2225*, and *AEQU_2224* (Figure 4B). All had a calculated Gibbs-free energy (ΔG) value of <–10 kcal/mol (Figure 4C), suggesting a strong transcription termination capacity. The position of these terminators agrees well with the gene expression patterns determined by RT-qPCR (the only exception being ORF *AEQU_2225*, for which no significant differences in the amount of transcripts were recorded with respect to the *AEQU_2224* and *AEQU_2223* ORFs). To confirm whether the predicted terminators related to different transcripts, the expression of the intergenic regions of all ORFs in the equol gene cluster was also analysed by RT-PCR (Figure 4B). Surprisingly, amplification was obtained for all intergenic regions, indicating that the operon was transcribed as a single RNA molecule of ~15 kbp long. Within the same expression patterns, genes located downstream of weak putative terminators identified (ΔG −7 to −8 kcal/mol) showed a little less transcription than those located upstream.

## 4. Discussion

A transcriptional analysis of the ORFs and intergenic regions of the equol gene cluster of *A. equolifaciens* DSM19450^T^ was undertaken in this work to identify the genetic elements involved in equol biosynthesis and its regulation. Although three reductase enzymes (daidzein-, dihydrodaidzein-, and tetrahydrodaidzein-reductase) have been described as essential in the conversion of daidzein to equol [34], few studies have examined the genetics and biochemistry of equol formation in equol-producing bacteria [26,29]. Proteomic analyses of *S. isoflavoniconvertens* grown with daidzein have shown the induction, in addition to the reductases, of five other proteins encoded by genes located upstream and downstream of those coding for these reductases [29]. Enzymes of this cluster are thought to be involved in equol production and might all be regulated in a coordinated manner [29].

Prior to starting the genetic analyses, the growth behavior of *A. equolifaciens* DSM19450^T^ was examined in the presence of daidzein. Compared to control cultures, the presence of daidzein induced greater bacterial growth. However, the maximum growth was reached at the lowest daidzein concentration tested (50 µM), suggesting that, as seen in other intestinal bacterial species [35,36], the growth of *A. equolifaciens* might be modulated by the daidzein and/or equol concentration. The growth curves for *A. equolifaciens* with daidzein were very similar to those reported for *S. isoflavoniconvertens* [30] and *E. mucosicola* [37]. Nonetheless, *A. equolifaciens* in GAM-Arg grew much more than *S. isoflavoniconvertens* [30] or *E. mucosicola* [37] in BHI medium, or *Slackia* sp. NATTS in GAM with 1% glucose [21], suggesting that gut-dwelling bacteria might obtain additional energy from arginine via the arginine dihydrolase pathway [38].

The production of equol by *A. equolifaciens* increased with the amount of daidzein present, suggesting this compound induces its own metabolism. The induction of enzymes involved in daidzein conversion to equol has already been reported for *E. mucosicola* [30] and *S. isoflavoniconvertens* [29]. Generally speaking, *A. equolifaciens* DSM19450^T^ metabolized most of the daidzein (>95%) present, but only under one third of this (27–29%) was converted into dihydrodaidzein. In contrast most dihydrodaidzein seems to be transformed into equol. All these metabolic steps occurred rather quickly, as the daidzein disappeared after just 10 h of incubation and the level of both dihydrodaidzein and equol remained constant thereafter. For other equol-producing bacteria; e.g., *A. celatus* do03 [19], *Eggerthella* spp. [39], *S. isoflavoniconvertens* [37], and *Slackia* NATTS [25], daidzein-to-equol conversion ratios ranging from 50 to 90% have been reported. *A. equolifaciens* DSM19450^T^ showed a daidzein-to-equol transformation ratio close to that reported for *A. celatus* (32%) [40]. The fact that all the daidzein disappeared but only a small amount was transformed into dihydrodaidzein strongly suggests that other daidzein-derived metabolites (not identified in this study) are also produced by *A. equolifaciens* DSM19450^T^. Indeed, the simultaneous production of equol and *O*-desmethylangolensin (*O*-DMA) has already been reported for *Eubacterium ramulus* Julong 601 [41]. The conversion of daidzein into novel, as yet un-detected derivatives, cannot be ruled out (the same has recently been reported for the conversion of genistein into 5-hydroxy-dehydroequol) [42].

The transcriptional analysis of the genes and their flanking intergenic sequences in the absence of daidzein revealed the low level constitutive expression of 13 genes in the *A. equolifaciens* cluster. The fact that the expression of most genes in the equol operon increased in the presence of daidzein further suggests that all are involved in its metabolism. Five genes within the cluster experienced similarly high induction; these genes coded for the dihydro- and tetrahydro-daidzein reductases, a dihydrodaidzein racemase, and the alpha and beta subunits of an electron flavoprotein (Supplementary Table S2). Beyond the reductases, other proteins of the cluster might be indispensable in, or have influence on, equol production. As such, a dihydrodaidzein racemase converting (*R*)-dihydrodaidzein into (*S*)-dihydrodaidzein has been demonstrated essential for efficient biosynthesis of equol in *L. garvieae* [43]. This protein is 48% homologous to that encoded by ORF *AEQU_2234* in *A. equolifaciens*. The flavoprotein in the cluster (*AEQU_2232* and *AEQU_2233*, alpha and beta subunits) might participate in the transfer of electrons required by the reductases (as demonstrated for other proteins of this family that funnel electrons into the electron transport chain) [44] or divert electrons from dehydrogenases to nitrogenases [45]). Recently, the structure of the daidzein reductase of *Eggerthella* was resolved to be a homo-octameric protein containing FMA, FAD and an aggregate of 4Fe-4S ions, all acting as cofactors [46]. The present results suggest that the ORFs surrounding the daidzein reductase gene (Supplementary Table S2; Supplementary Figure S1), all of which had a similar expression pattern, might encode proteins involved in the synthesis of some of the above cofactors. Still other genes might encode a two-component response regulator and glutamate synthase and dehydrogenase enzymes homologous to components of the isoflavone-induced NodVW system in *Bradyrhizobium japonicum* [47,48]. These proteins might detect and react to the presence of daidzein in the environment. Finally, genes encoding proteins reported to act as [Fe-S]-maturases (HydE enzymes in *Asaccharobacter* and *Adlercreutzia* [49]) might be responsible for the maturation of the flavine-dehydrogenase, as it contains an iron-sulphur binding domain (Supplementary Table S2; Supplementary Figure S1).

Since no information on transcription termination in *A. equolifaciens* or other equol-producing species is currently available, an in silico search for terminator-like sequences was performed to help elucidate the organization and underlying regulation mechanisms within the equol biosynthesis gene cluster. Among the eight putative terminator sequences identified, four concurred with those proposed in *S. isoflavoniconvertens* [29]. The Gibbs free-energy values of the terminators correlated well with the different expression patterns determined by RT-qPCR. However, surprisingly, the transcriptional analysis of all intergenic regions revealed the whole operon to be transcribed as a single unit. This suggests that internal promoters/terminators within the cluster (producing shorter transcripts) might also exist, as recently demonstrated in operons from other bacteria [50]. These signals and/or other post-transcriptional regulatory circuits that modulate the efficiency of mRNA translation into protein might shape the production of equol by *A. equolifaciens* DSM19450^T^.

## 5. Conclusions

*A. equolifaciens* DSM19450^T^ transforms daidzein in its growth medium into equol. However, only one third part of the daidzein added is converted into dihydrodaidzein and then into equol. The expression of the 13 contiguous genes tested was enhanced when the bacterium was incubated with daidzein, suggesting the expression of all these genes to be modulated by this isoflavone. Although the cluster was translated into a single RNA molecule, the genes were expressed under four distinct expression patters, each under the control of different putative promoter and terminator signals. More research will be needed to discover all the elements of the daidzein metabolism pathways in *A. equolifaciens* and their involvement in equol production. This knowledge is thought to be pivotal in attaining large-scale biotechnological production of equol using this bacterium or its genetic machinery, and for designing strategies aimed at increasing its endogenous production.

## Supplementary Materials

The following are available online at https://www.mdpi.com/2072-6643/11/5/993/s1, Figure S1: Comparison of equol biosynthesis clusters from *Slackia isoflavoniconvertens* DSM22006T and *A. equolifaciens* DSM19450T. In *S. isoflavoniconvertens*, green-colored genes have been demonstrated to be essential for equol production; proteins from orange-colored genes have been found to be induced by daidzein; Table S1: Annotation of the open reading frames (*orfs*) within the equol biosynthesis gene cluster of *A. equolifaciens* DSM19450^T^; Table S2: Sequence, product size and annealing temperature of PCR primers used in gene expression analysis by RT-PCR; Table S3: Sequence, product size and annealing temperature of primers used to study gene expression of intergenic regions by RT-PCR; Table S4: Accuracy, efficiency and regression equation obtained for the amplification of equol cluster genes with the primers designed in this study for RT-qPCR analysis.
