# Supplementary material for: Transcriptional Regulation of the Equol Biosynthesis Gene Cluster in Adlercreutzia equolifaciens DSM19450T
Source: Nutrients. 2019 Apr 30;11(5):993. doi: 10.3390/nu11050993 (PMC6566806; doi:10.3390/nu11050993)
Supplement: Supplementary file 1 [file nutrients-11-00993-s001.zip › Table 2 supplementary material-primers RT-PCR.docx]

**Supplementary Table 2.-** Sequence, product size and annealing temperature of PCR primers used in gene expression analysis by RT-PCR.

| **Gene** | **Primers** | **Secuencia 5´ - 3´** | **Product length (bp)** | **Annealing temperature**  **(ºC)** |
| --- | --- | --- | --- | --- |
| ***AEQU_2235*** | 35f | GAGTTGGCATCATGATCTGCGATA | 515 | 55 |
|  | 35r | CGTCCATCAACAGGCAGAGAATCT |  |  |
| ***AEQU_2234*** | 34f | CGAGACCATGAGGGATTTCAACGA | 401 | 55 |
|  | 34r | GCGTACAGGTCGATGCCCATGTC |  |  |
| ***AEQU_2233*** | 33f | GGTGACGAGGTGGTTGGTTTGT | 531 | 55 |
|  | 33r | GGCAGGATCGGATCCATCGAACA |  |  |
| ***AEQU_2232*** | 32f | GCTGTCGCTTCGTCGGTCAGCAA | 456 | 68 |
|  | 32r | CCGAAATACCCACCGACACGTAGA |  |  |
| ***tdr*** | tdrf | GATACCATCGATTTCCTCAAGGAT | 660 | 62 |
|  | tdrr | CGTTCTCAGCAAGACGGTCGATGT |  |  |
| ***ddr*** | ddrf | CTGGGCAAGCGATTGGAAGGTAA | 651 | 65 |
|  | ddrr | GATCGATAGCCTGCTGGGTGGTCT |  |  |
| ***AEQU_2229*** | 29f | CATTGGATTCGGAAGAGTTGTCA | 502 | 55 |
|  | 29r | CAGGATCGAAGCCACTGATGGCTT |  |  |
| ***dzr*** | dzrf | GACCATCGAGGAGATTCACGAGTT | 702 | 55 |
|  | dzrr | CTCGCGATCGAACTGGTACAGTGT |  |  |
| ***AEQU_2227*** | 27r | GCAACATGACCATCGACTTCACCA | 537 | 62 |
|  | 27f | GGATGGTGCTTGAAGTCTTCCAT |  |  |
| ***AEQU_2226*** | 26r | CACCTTGCCGAAGATCACGGTGAA | 255 | 60 |
|  | 26f | CGACACGATGGTCTCCTCGCAGCA |  |  |
| ***AEQU_2225*** | 25r | GGTGAAGATGATCCGTAAGGACAA | 763 | 55 |
|  | 25f | GAGCCTGCAGGCACATCATCTCGA |  |  |
| ***AEQU_2224*** | 24r | CGTTAACATTCCCACGCTGTGCT | 596 | 55 |
|  | 24f | GCCACCTGCACCACGGTCTCCAC |  |  |
| ***AEQU_2223*** | 23r | CGAGGAAGGGTGGCAGGCAGGCTT | 615 | 68 |
|  | 24r | CGACAAGTTCGGCATCACGACGTT |  |  |
