# Supplementary material for: Transcriptional Regulation of the Equol Biosynthesis Gene Cluster in Adlercreutzia equolifaciens DSM19450T
Source: Nutrients. 2019 Apr 30;11(5):993. doi: 10.3390/nu11050993 (PMC6566806; doi:10.3390/nu11050993)
Supplement: Supplementary file 1 [file nutrients-11-00993-s001.zip › Table 3 supplementary material-primers RT-PCR intergenic.docx]

| **Intergenic region** | **Primers** | **Sequence (5’ - 3’)** | **Amplicon size (bp)** | **Annealing temperature (ºC)** |
| --- | --- | --- | --- | --- |
| 2235-2234^a^ | 35-34-F | GAATCGTGTGCAGGCTGCTG | 332 | 60ºC |
|  | 35-34-R | CAGGTCGTTGAAATCCCTCA |  |  |
| 2234-2233 | 34-33-F | CAACATCGAAGTGGCTCTCA | 283 | 60ºC |
|  | 34-33-R | GCACAAACCAACCACCTCGT |  |  |
| 2233-2232 | 33-32-F | GTTCGATGGATCCGATCCTG | 375 | 60ºC |
|  | 33-32-R | GAACACGGGAGGCTGCAGCA |  |  |
| 2232-tdr | 32-31-F | CGACTTGAACAAGGTGGTT | 286 | 60ºC |
|  | 32-31-R | CTTCTGCTCGCTCGACTCGA |  |  |
| *tdr-ddr* | 31-30-F | GCAATATGCAGGTAGTCGACA | 348 | 60ºC |
|  | 31-30-R | GCTTACCTTCCAATCGCTTG |  |  |
| *ddr*-2229 | 30-29-F | GACCACCCAGCAGGCTATCGA | 382 | 60ºC |
|  | 30-29-R | CGAACCAAGCCTCGATGACA |  |  |
| 2229-*dzr* | 29-28-F | GCAACATGCTGATCAACAAC | 340 | 60ºC |
|  | 29-28-R | CCATGGGCTGTCGCACGATG |  |  |
| *dzr*-2227 | 28-27-F | CGACGACATCGAGCAGATT | 378 | 60ºC |
|  | 28-27-R | CCGTTGGCATACTTGTCGA |  |  |
| 2227-2226 | 27-26-F | CAGCAATCTGACGAAGCGTA | 235 | 60ºC |
|  | 27-26-R | CTTCACCAGCTTCAGGAACT |  |  |
| 2226-2225 | 26-25-F | CATCTGCTGCGAGGAGACCA | 359 | 60ºC |
|  | 26-25-R | GCCTCGACGAACGCTCCCAT |  |  |
| 2225-2224 | 25-24-F | CTGCCTGCGATGCGATGTGT | 182 | 60ºC |
|  | 25-24-R | GATGCAGTTCAGATCCTTCA |  |  |
| 2224-2223 | 24-23-F | CCGACATTGCGAAGCTGTAC | 396 | 60ºC |
|  | 24-23-R | GCAGATGTTCGTGAACTCGA |  |  |

**Table 3 Supplementary material.** Sequence, product size and annealing temperature of primers used to study gene expression of intergenic regions by RT-PCR.

^a^Numbers of the intergeneric regions refer to ORF numbers (AEQU_) or to their corresponding genes.
