# Supplementary material for: Transcriptional Regulation of the Equol Biosynthesis Gene Cluster in Adlercreutzia equolifaciens DSM19450T
Source: Nutrients. 2019 Apr 30;11(5):993. doi: 10.3390/nu11050993 (PMC6566806; doi:10.3390/nu11050993)
Supplement: Supplementary file 1 [file nutrients-11-00993-s001.zip › Table 4 supplementary material-primers RT-qPCR.docx]

| **Gen** | **Primer** | **Sequence (5´-3´)** | **R²** | **Regression equation** | **Effiency** |
| --- | --- | --- | --- | --- | --- |
| ***AEQU_2235*** | Aequ_2235.qPCR-F | CGCCTGAATCGTTCACTTCA | 0.9973 | y = -3.3346x + 24.304 | 99.47% |
|  | Aequ_2235.qPCR-R | GTGTCGCTGTGCTGGTTCAA |  |  |  |
| ***AEQU_2234*** | Aequ_2234.qPCR-F | GTACTTCGAGCACAACCTGATGA | 0.9979 | y = -3.3234x + 27.444 | 99.93% |
|  | Aequ_2234.qPCR-R | CGTTGAGAGCCACTTCGATGT |  |  |  |
| ***AEQU_2233*** | Aequ_2233.qPCR-F | CGCACGGTGGTGTTGGA | 0.9997 | y = -3.3572x + 26.562 | 98.54% |
|  | Aequ_2233.qPCR-R | TTCAACACCCTTGATTTCCTCAA |  |  |  |
| ***AEQU_2232*** | Aequ_2232.qPCR-F | CGGTGCCAAGACCATCGT | 0.9988 | y = -3.5962x + 29.539 | 100.55% |
|  | Aequ_2232.qPCR-R | GCAGGAACCACCTTGTTCAAG |  |  |  |
| ***tdr*** | Aequ_2231.qPCR-F | CAGCCACTATCGCGCAAACT | 0.9986 | y = -3.2687x + 27.398 | 102.27% |
|  | Aequ_2231.qPCR-R | GATCCTTGAGGAAATCGATGGT |  |  |  |
| ***ddr*** | Aequ_2230.qPCR-F | CCACCCAGCAGGCTATCG | 0.9971 | y = -3.255x + 27.107 | 102.87% |
|  | Aequ_2230.qPCR-R | CAGGTTGACCGCCGTGAT |  |  |  |
| ***AEQU_2229*** | Aequ_2229.qPCR-F | TCGGCACGATCATTTCGTT | 0.9988 | y = -3.327x + 27.245 | 99.78% |
|  | Aequ_2229.qPCR-R | GACAAAGCCGCCGAACAC |  |  |  |
| ***dzr*** | Aequ_2228.qPCR-F | GGCCGTCTGGGCAAGTACTA | 0.9985 | y = -3.3057x + 28.073 | 100.68% |
|  | Aequ_2228.qPCR-R | GGTCTCCGGCGTGGCATT |  |  |  |
| ***AEQU_2227*** | Aequ_2227.qPCR-F | GAGTCGGCTGGATCGAAGAAC | 0.9996 | y = -3.2753x + 26.722 | 101.98% |
|  | Aequ_2227.qPCR-R | TTGGCATACTTGTCGAAAACCTT |  |  |  |
| ***AEQU_2226*** | Aequ_2226.qPCR-F | ATCACGGTGAACGTCGATGA | 0.9948 | y = -3.2587x + 28,659 | 102.70% |
|  | Aequ_2226.qPCR-R | GCCGTGAAGCCGATATGC |  |  |  |
| ***AEQU_2225*** | Aequ_2225.qPCR-F | GTCGACCGCACCTATTTCGT | 0.9898 | y = -3.3613x + 28.935 | 98.38% |
|  | Aequ_2225.qPCR-R | TGGCCATGATGACGGTCTT |  |  |  |
| ***AEQU_2224*** | Aequ_2224.qPCR-F | CTTCGACCGCGTCTTCGA | 0.9984 | y = -3.4123x + 28.964 | 96.36% |
|  | Aequ_2224.qPCR-R | CCCAGGAACTCCACGAACTC |  |  |  |
| ***AEQU_2223*** | Aequ_2223.qPCR-F | CGCCAACGACTGCCACTAC | 0.9917 | y = -3.3375x + 28.938 | 99.35% |
|  | Aequ_2223.qPCR-R | CAGAATCTGCTCACCGGTAAGG |  |  |  |
| ***tuf*** | Aequ_Tuf.qPCR-F | CACGCCGACTACGTGAAGAAC | 0.9933 | y = -3.3272x + 28.897 | 99.78% |
|  | Aequ_Tuf.qPCR-R | CCGTCGGTAGCAGCGATAAC |  |  |  |
| ***gadpdh*** | Aequ_gadpdh.qPCR-F | AGCACGTGACCATGCTTTCC | 0.9982 | y = -3.3168x + 27.253 | 100.21% |
|  | Aequ_gadpdh.qPCR-R | GGCCTCTACGACCACATCCA |  |  |  |

**Table 4 Supplementary material.** Accuracy, efficiency and regression equation obtained for the amplification of equol cluster genes with the primers designed in this study for RT-qPCR analysis.
