# Supplementary material for: Transcriptional Regulation of the Equol Biosynthesis Gene Cluster in Adlercreutzia equolifaciens DSM19450T
Source: Nutrients. 2019 Apr 30;11(5):993. doi: 10.3390/nu11050993 (PMC6566806; doi:10.3390/nu11050993)
Supplement: Supplementary file 1 [file nutrients-11-00993-s001.zip › SuplementaryFigure1.pdf]

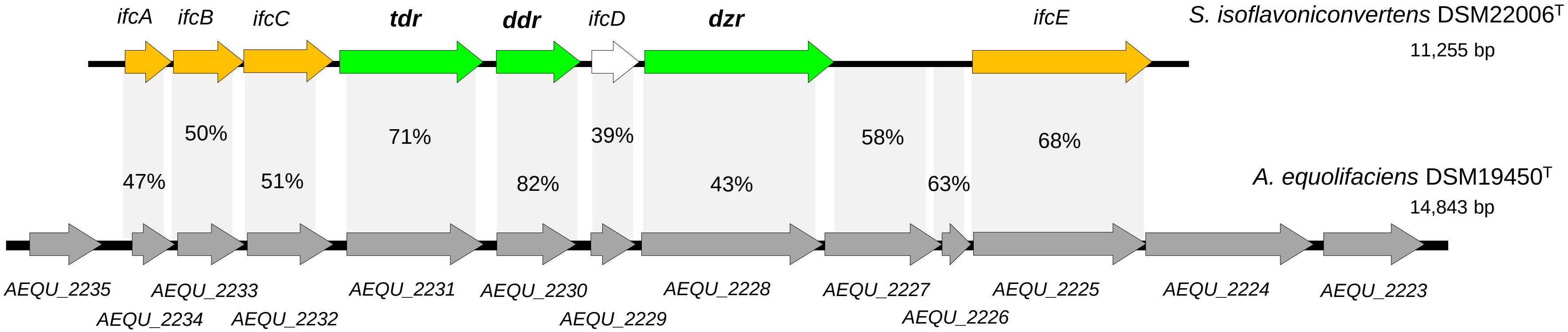

Supplementary Figure 1.- Comparison of equol biosynthesis clusters from *Slackia isoflavoniconvertens* DSM22006^{T} and *A. equolifaciens* DSM19450^{T}. In *S. isoflavoniconvertens*, green-coloured genes have been demonstrated to be essential for equol production; proteins from orange-coloured genes have been found to be induced by daidzein (Schöder et al., 2013).
