# Supplementary material for: Transcriptional Regulation of the Equol Biosynthesis Gene Cluster in Adlercreutzia equolifaciens DSM19450T
Source: Nutrients. 2019 Apr 30;11(5):993. doi: 10.3390/nu11050993 (PMC6566806; doi:10.3390/nu11050993)
Supplement: Supplementary file 1 [file nutrients-11-00993-s001.zip › Table 1 supplementary material-Orfs.docx]

| **Locus_tag** | **Strand** | **Position^a^** | **Gene product** |
| --- | --- | --- | --- |
|  |  |  |  |
| *AEQU_2235* | - | 2791670-2792440 | Two-component response regulator |
| *AEQU_2234* | - | 2791008-2791460 | Putative dihydrodaizein racemase |
| *AEQU_2233* | - | 2790267-2790986 | Electron transfer flavoprotein beta subunit |
| *AEQU_2232* | - | 2789323-2790237 | Electron transfer flavoprotein alpha subunit |
| *AEQU_2231* | - | 2787796-2789259 | Putative tetrahydrodaidzein reductase |
| *AEQU_2230* | - | 2786868-2787716 | Putative dihydrodaidzein reductase |
| *AEQU_2229* | - | 2786295-2786774 | Hypothetical protein |
| *AEQU_2228* | - | 2784304-2786232 | Putative daidzein reductase |
| *AEQU_2227* | - | 2782920-2784233 | Flavin-dependent dehydrogenase |
| *AEQU_2226* | - | 2782612-2782923 | Putative ferredoxin |
| *AEQU_2225* | - | 2780557-2782395 | Putative glutamate synthase |
| *AEQU_2224* | - | 2778770-2780563 | Dehydrogenase |
| *AEQU_2223* | - | 2777598-2778668 | Conserved hypothetical protein |
|  |  |  |  |

**Supplementary Table 1.-** Annotation of the open reading frames (*orfs*) within the equol biosynthesis gene cluster of *A. equolifaciens* DSM19450^T^.

^a^In *Adlercreutzia equolifaciens* DSM19450^T^ genome (GenBank Accession no.: GCA_000478885.1).
